# Draft Genome Perspective of *Staphylococcus saprophyticus* Strain SU8, an *N*-Acyl Homoserine Lactone-Degrading Bacterium

**DOI:** 10.1128/genomeA.01097-15

**Published:** 2015-09-24

**Authors:** Kok-Gan Chan, Joanita Sulaiman, Delicia Ann Yong, Kok Keng Tee, Wai-Fong Yin, Kumutha Priya

**Affiliations:** a Division of Genetics and Molecular Biology, Institute of Biological Sciences, Faculty of Science, University of Malaya, Kuala Lumpur, Malaysia; b Institute for Public Health, National Institutes of Health Malaysia, Kuala Lumpur, Malaysia; c Department of Medicine, Faculty of Medicine, University of Malaya, Kuala Lumpur, Malaysia

## Abstract

*Staphylococcus saprophyticus* strain SU8 was isolated from a pristine water source in Malaysia and it exhibited degradation of *N-*hexanoylhomoserine lactone. Here we report the draft genome sequence of *S. saprophyticus* strain SU8 to further understand its quorum quenching abilities.

## GENOME ANNOUNCEMENT

*Staphylococcus saprophyticus*, belonging to the genus of *Staphylococcus*, is a Gram-positive, nonmotile, non-spore-forming, and coagulase-negative bacterium (1). *S. saprophyticus* is the second most common cause of urinary tract infections (UTIs) after *Escherichia coli* (2, 3). The quorum-sensing (QS) system *agr* has been discovered in *Staphylococcus aureus* and *Staphylococcus epidermidis* and is well known to control the expression of toxins, virulence factors, and biofilm formation (4). Also, new classes of quorum-quenching molecules named yayurea A and yayurea B, which inhibit the expression of QS-controlled genes in Gram-negative bacteria, were isolated from *Staphylococcus delphini* (5). We previously reported a lactonase gene in *Staphylococcus* sp. strain AL1 that can degrade the QS molecules, namely, the *N*-acyl homoserine lactones (AHLs) (6). Hence, the draft genome of *S. saprophyticus* strain SU8 will enable further understanding of its quorum- quenching potential and mechanism.

In the present work, *S. saprophyticus* strain SU8 was isolated from a pristine water source in Malaysia. The strain was isolated upon several enrichment transfers in *N*-hexanoyl homoserine lactone (C6-HSL) containing KG medium as the sole source of carbon and nitrogen (7). An AHL-degradation assay showed that this strain was capable of C6-HSL degradation (8).

The genomic DNA of *S. saprophyticus* strain SU8 was extracted and purified using the MasterPure Complete DNA purification kit (Epicentre, Inc., USA) per the manufacturer’s protocol. Purity and concentration of the genomic DNA were assessed by NanoDrop spectrophotometer (Thermo Scientific, USA) and Qubit version 2.0 fluorometer (Life Technologies, USA), respectively. Normalized paired-end libraries were prepared using the Nextera DNA library preparation kit (Illumina, CA, USA) and whole-genome sequenced on the Illumina MiSeq (Illumina) platform. Low-quality sequence reads (cutoff value of 0.1), ambiguous nucleotides, and sequence lengths of less than 50 nucleotides were trimmed prior to sequence assembly. The genome sequence was trimmed and assembled using CLC Genomics Workbench version 7.5 (CLC Bio, Denmark). Genome annotation was done using Prokka (9).

The whole-genome sequencing of *S. saprophyticus* strain SU8 resulted in 466,132 paired-end reads with an average read length of 182.07 bp. The genome assembly yielded 44 contigs with an average coverage of approximately 31× and *N*_50_ of 236 kbp. The final draft genome sequence of *S. saprophyticus* strain SU8 contained 2,708,421 bases with a G+C content of 33.0%. Gene annotation predicted 2,674 genes with 2,537 protein coding sequences. A total of 57 tRNAs as well as 5 copies of rRNAs were predicted. Of these, one gene encoding AHL lactonase was found in contig 4 of the genome sequence by using the BLASTx function against the quorum quenching lactonase database obtained from UniProtKB Protein Knowledge.

### Nucleotide sequence accession numbers.

This whole-genome shotgun project has been deposited at DDBJ/EMBL/GenBank under the accession number JXBG00000000. The version described in this paper is the first version, JXBG01000000.

## References

[1] Schleifer KH, Kloos WE 1975 Isolation and characterization of *Staphylococci* from human skin I. Amended descriptions of *Staphylococcus epidermidis* and *Staphylococcus saprophyticus* and descriptions of three new species: *Staphylococcus cohnii*, *Staphylococcus haemolyticus*, and *Staphylococcus xylosus*. Int J Syst Bacteriol 25:50–61. doi:10.1099/00207713-25-1-50.

[2] Ronald A 2003 The etiology of urinary tract infection: traditional and emerging pathogens. DIS Mon 49:71–82. doi:10.1067/mda.2003.8. 12601338

[3] Birgitta H, Mãrdh PA 1983 *Staphylococcus saprophyticus* as a common cause of urinary tract infections. Clin Infect Dis 6:328–337. 10.1093/clinids/6.3.328 6377440

[4] Kong KF, Vuong C, Otto M 2006 *Staphylococcus* quorum sensing in biofilm formation and infection. Int J Med Microbiol 296:133–139. doi:10.1016/j.ijmm.2006.01.042. 16487744

[5] Chu YY, Nega M, Wölfle M, Plener L, Grond S, Jung K, Götz F 2013 A new class of quorum quenching molecules from *Staphylococcus* species affects communication and growth of Gram-negative bacteria. PLoS Pathog 9:e1003654. doi:10.1371/journal.ppat.1003654. 24098134 PMC3784491

[6] Chong TM, Tung HJ, Yin WF, Chan KG 2012 Insights from the genome sequence of quorum-quenching *Staphylococcus* sp. strain AL1, isolated from traditional Chinese soy sauce brine fermentation. J Bacteriol 194:6611–6612. doi:10.1128/JB.01669-12. 23144375 PMC3497501

[7] Chan KG, Yin WF, Sam CK, Koh CL 2009 A novel medium for the isolation of *N-*acylhomoserine lactone-degrading bacteria. J Ind Microbiol Biotechnol 36:247–251. doi:10.1007/s10295-008-0491-x. 18946694

[8] Chong TM, Koh CL, Sam CK, Choo YM, Yin WF, Chan KG 2012 Characterization of quorum sensing and quorum quenching soil bacteria isolated from Malaysian tropical montane forest. Sensors 12:4846–4859. doi:10.3390/s120404846. 22666062 PMC3355444

[9] Seemann T 2014 Prokka: rapid prokaryotic genome annotation. BioInformatics 30:2068–2069. doi:10.1093/bioinformatics/btu153. 24642063

